# Conservative Management of Intraperitoneal Bladder Injury During the Transurethral Resection of a Bladder Tumor: A Case Report

**DOI:** 10.7759/cureus.81703

**Published:** 2025-04-04

**Authors:** Turki Alghamdi, Ali R Al Zaid, Mohammed Almomen, Murtadha Alnemer, Abdulaziz Alhussaini

**Affiliations:** 1 Urology, King Fahad Specialist Hospital, Dammam, SAU; 2 Urology, Dammam Medical Complex, Dammam, SAU

**Keywords:** bladder perforation, conservative medical management, iatrogenic bladder injury, intraperitoneal bladder perforation, transurethral resection of bladder tumor (turbt)

## Abstract

Bladder injury can be intraperitoneal or extraperitoneal due to multiple mechanisms, with surgical repair as the mainstream management in intraperitoneal injuries. We present a case of iatrogenic intraperitoneal bladder injury during transurethral resection of a bladder tumor located in the superior bladder wall. Many cases reported the role of conservative management of intraperitoneal injuries that included intraperitoneal drain, urethral catheterization, and continued monitoring of vital signs and serial abdominal examination. Still, conservative management of intraperitoneal bladder injury is not established yet, with a clinically accepted role in many cases reported.

## Introduction

Bladder injury can be caused by penetrating or blunt trauma to the abdominal wall or pelvis or due to iatrogenic causes during urological, gynecological, and colorectal surgeries. Additionally, it is classified into extraperitoneal and intraperitoneal injuries, with an incidence of bladder injury being 60% and 30%, respectively. The remaining was caused by mixed intra- and extraperitoneal injuries [1]. Extraperitoneal bladder injury is mostly related to concomitant pelvic fracture. Nevertheless, intraperitoneal bladder injury is often associated with blunt trauma to the dome of the bladder, which can lead to leakage of urine into the peritoneum, resulting in complications such as sepsis, chemical ileus, and peritonitis [2]. Extraperitoneal bladder injury is mostly managed conservatively by using a urethral Foley catheter without intraoperative surgical repair. Conversely, most intraperitoneal bladder injuries require surgical exploration for bladder wall repair [3]. However, some evidence suggests that minimal bladder injury could be treated conservatively in selected cases, considering patient stability, and no sign of intraperitoneal infection [4,5]. In this study, we present the case of an iatrogenic intraperitoneal bladder injury during transurethral resection of a bladder tumor (TURBT) that was managed conservatively without further surgeries.

## Case presentation

A 58-year-old male presented with a medical history of diabetes mellitus, hypertension, dyslipidemia, and benign prostatic hyperplasia on tamsulosin and finasteride with recurrent episodes of urinary. The patient was planned for transurethral resection of the prostate (TURP). On diagnostic urethrocystoscopy, there were kissing lateral lobes and an enlarged median lobe with a high bladder neck. During bladder inspection, we found a small bladder lesion around 1x1 cm in size located at the superior bladder wall, raising suspicion for malignancy. A biopsy of the lesion was taken using cup biopsy forceps, and resection of the median lobe started. However, due to bleeding and lack of vision, the procedure was aborted, and a three-way urethral catheter was inserted with continuous bladder irrigation (CBI). The patient was stable post-operatively and thus was discharged home, and he consented to and was booked for a second TURP and TURBT after one week as the bladder biopsy showed benign pathology.

In the second operation, we started with the excision of the bladder lesion, which was confined to the mucosa of the superior bladder wall; the resection was superficial with a semi-distended bladder and under general anesthesia. After that, prostate resection and the resection of the left lateral lobe were completed. The patient developed marked abdominal distention, and thus the procedure was aborted. Hemostasis was secured, and the three-way urethral catheter was inserted with CBI.

**Figure 1:**
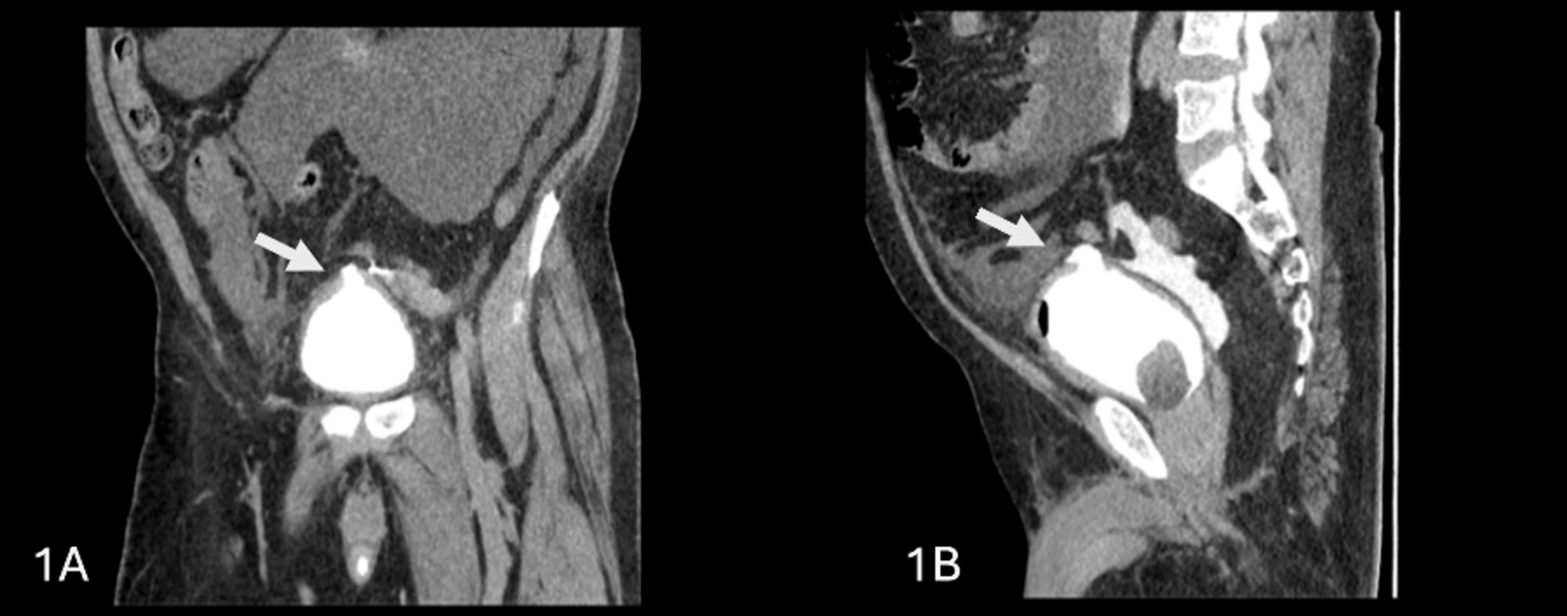
(A) Coronal view of computed tomography cystogram showing superior bladder wall defect with extravasation of intravesical contrast. (B) Sagittal view.

Postoperatively, the patient exhibited signs of hemodynamic instability and abdominal tenderness. A computed tomography (CT) scan of the abdomen and pelvis confirmed intraperitoneal extravasation of contrast material and a bladder wall defect, consistent with intraperitoneal bladder rupture (Figures 1A, 1B). As an immediate action, the patient was admitted to the intensive care unit (ICU) for close monitoring. Initial management included nothing per oral, nasogastric tube insertion, intravenous fluids, and broad-spectrum antibiotics. Since the perforation was small, we intended a trial of non-operative management with a placement of a urethral catheter for urinary drainage and an abdominal drain to prevent urinary ascites. An interventional radiology (IR) procedure was performed to place a percutaneous drain in the right iliac fossa, which yielded around 7 liters of bloody fluid out of the drain.

The condition of the patient improved clinically after the insertion of the intraperitoneal drain. The peritoneal drain output was around 7,620 mL of bloody fluid, and the urethral catheter output in CBI was 1,500 mL with mild hematuria. On the second postoperative day, the patient's condition improved, with stable vital signs and resolution of abdominal distention. The peritoneal drain output decreased to 25 mL with hemoserous output, and urethral catheter output on CBI was clear. Laboratory wise, the patient maintained a normal hemoglobin level of 14.8 g/dL, with normal renal functions and balanced electrolytes. Subsequently, the patient was transferred to the surgical ward for continued observation. The peritoneal drain was removed on the sixth postoperative day following minimal drainage. The patient's clinical condition remained stable, and he was discharged home with a urethral catheter and instructions for follow-up.

**Figure 2:**
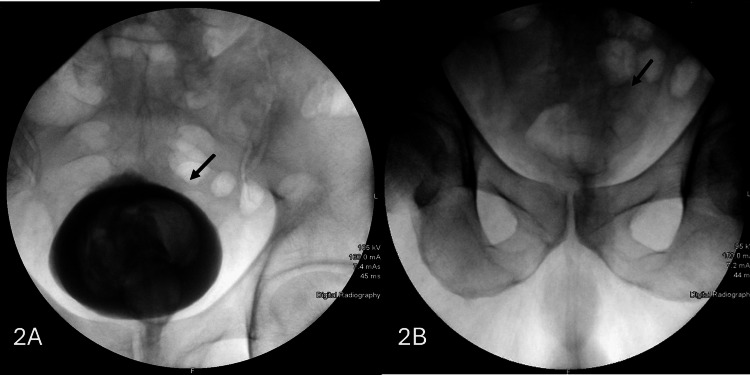
X-ray cystogram during (A) the filling phase with no extravasation and (B) during the voiding phase.

An X-ray cystogram was performed two weeks after bladder injury, which did not reveal any evidence of extravasation of intravesical contrast (Figures 2A, 2B). Six weeks after the initial surgery, the patient reported no complaints, and the histopathological report of bladder tissue showed benign pathology.

## Discussion

It has been believed that intraperitoneal bladder injuries require surgical repair. Among 1,570 patients who underwent TURBT, there was a risk of intraperitoneal bladder perforation in around 5%, and all intraperitoneal perforations were managed with surgical correction [6]. However, our approach was to manage the patient conservatively as long as he was clinically responding and to go for open repair if conservative management failed to allow bladder wall healing or the patient developed signs of chemical peritonitis. In the literature, many cases reported the role of conservative management in different mechanisms of the intraperitoneal bladder. Geng et al. reported conservative management that includes urethral catheterization and peritoneal drain in blunt abdominal trauma [7]. Moreover, insertion of urethral catheter and peritoneal drain was reported as successful conservative management in a case of intraperitoneal bladder perforation post-cesarean section [8]. TURBT is also associated with bladder injury. Pansadoro et al. reported non-operative management of intraperitoneal bladder injury during TURBT [9]. In our case, the identification of intraperitoneal bladder injury was immediately after TURBT with abdominal distention. Computed tomography (CT) cystogram was the diagnostic modality in most cases reported, with extravasation of the contrast into the intraperitoneal space as a diagnostic sign for intraperitoneal bladder injury [7-9]. Patient stability and small bladder perforation in CT cystogram made the conservative management more likely. Paracentesis with analysis of fluid creatinine is a diagnostic and therapeutic intervention in conservative management.

## Conclusions

Small intraperitoneal bladder perforations could be managed conservatively, deferring an invasive repair approach, in carefully selected settings. Continuous monitoring of vital signs, serial abdominal examinations, urethral catheterization, and abdominal drain are the mainstreams of conservative management in bladder perforation. Follow-up with a cystogram after patient improvement is recommended. Conservative management in appropriate clinical indications prevents complications of an invasive approach and decreases hospitalization.
